# Saving Strength

**Published:** 1871-09

**Authors:** 


### SAVING STRENGTH.

After a night of good, sound, refreshing sleep, we rise from our beds with renewed strength and vigor. Multitudes go to bed almost utterly exhausted, but in many, very many cases, without having accomplished much, because the stock of strength which they had in the morning was not judiciously expended. The first work of the day should be done with great deliberation; then the muscles of the body gradually warm up and move easily and naturally. A horse will not travel far if he begins his journey in a gallop. That minister soon becomes exhausted who begins his sermon in a loud key. Any man who has a heavy day's work before him, should save all his strength for that work. The mechanic should not have to walk a long distance to his work, the farmer to his field, nor the rower to the water's edge. L. Sullivan has a farm in Lexington County, Illinois, of over forty thousand acres; it is just eight miles square. He has fifteen thousand acres this year under plough. Twenty years ago he gave a dollar and a half an acre for this farm; it is now, with what is on it, worth two millions of dollars. This tract is divided into thirty-two farms, and the whole moves on with the regularity of clock-work. Everything is systematized. He manages to get more work out of his men than any other man in all that region. In the first place, he pays them well and punctually; that makes them anxious to keep their places. In the second place, none are employed who drink spirituous liquors; they are required to keep cleanly, tidy homes, and are at all times treated with the courtesy and consideration which belongs to the highest type of man — the honest day laborer. But one other thing Mr. Sullivan does, for he is a philosopher. He does not allow his men to walk a mile or two to their working places — they ride; hence no strength is expended except on the work in hand. Perhaps the time may come when the farmer and the mechanic will find profit in studying the physiology of the human frame, its laws, and its needs. The time is rapidly approaching when it shall no longer be said that any fool can cultivate a farm.

